# Drought Responses in Mature European Beech Forests Vary Along Regional Environmental Gradients and Differ Among Admixed Tree Species

**DOI:** 10.1111/gcb.71097

**Published:** 2026-09-11

**Authors:** Mathias Mayer, Klaus Dolschak, Emilia Winter Artusio, Michael Grabner, Michael Tatzber, Iftekhar U. Ahmed, Elisabeth Wächter, Isolde K. Berger, Pétra Berger, Wolfgang Wanek, Torsten W. Berger

**Affiliations:** ^1^ Department of Ecosystem Management, Climate and Biodiversity, Institute of Forest Ecology BOKU University Vienna Austria; ^2^ Forest and Soil Ecology Swiss Federal Institute for Forest, Snow and Landscape Research (WSL) Birmensdorf Switzerland; ^3^ Department of Natural Sciences and Sustainable Resources, Institute of Wood Technology and Renewable Materials BOKU University Tulln an der Donau Austria; ^4^ Department of Forest Protection Austrian Research Centre for Forests (BFW) Vienna Austria; ^5^ Division of Terrestrial Ecosystem Research, Centre for Microbiology and Environmental Systems Science University of Vienna Vienna Austria

## Abstract

Climate change is increasing drought frequency and intensity, yet the drivers of variability in tree drought responses remain incompletely understood. We analyzed drought responses of *Fagus sylvatica* and seven admixed tree species across 62 forest stands in the Vienna Woods (Austria) using tree‐ring width, stable carbon isotopes (δ^13^C), and tree‐ring calcium concentrations. Drought resistance and resilience of *Fagus sylvatica* stands increased along a regional climate gradient, indicating smaller growth reductions and a return to pre‐drought growth levels at warmer, drier sites, whereas stands at cooler, moister sites frequently remained below pre‐drought growth levels. In addition, drought resistance decreased with increasing stand productivity and foliar nutrient status, indicating greater growth reductions in productive, nutrient‐rich stands. Tree‐ring δ^13^C and calcium concentrations increased during drought, indicating physiological stress; however, neither parameter explained spatial variation in growth reductions, consistent with a potential decoupling between short‐term physiological responses and longer‐term growth dynamics. Admixed species exhibited contrasting drought‐response strategies. *Quercus petraea* and *Quercus cerris* showed stronger growth reductions but returned more rapidly to pre‐drought levels than *Fagus sylvatica* . *Pinus nigra* and *Larix decidua* returned to or exceeded pre‐drought growth levels, with *Larix decidua* showing post‐drought overcompensation. These findings suggest that mixed forests comprising species with contrasting drought‐response strategies may enhance resilience under future drought regimes.

## Graphical abstract

Drought responses of European beech varied along regional environmental gradients, with greater resistance and resilience at warmer, drier sites. Tree‐ring δ^13^C and Ca increased during drought but were not related to growth responses. Contrasting responses among admixed species highlight the potential resilience benefits of mixed forests.

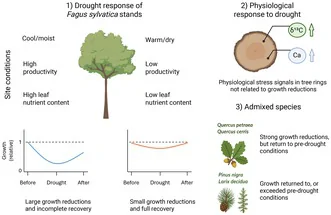

## 1 Introduction

Climate change is expected to increase the frequency, duration, and intensity of drought events across many forest regions worldwide, posing a major threat to forest productivity and stability (Allen et al. 2015; IPCC 2023). In Europe, recent extreme droughts have already caused widespread growth reductions, increased tree mortality, and shifts in species composition across forest ecosystems (Bréda et al. 2006; Schuldt et al. 2020; Senf and Seidl 2021). In particular, the severe drought events of 2003 and 2018–2019 led to pronounced declines in tree vitality, crown damage, and mortality across large parts of Central Europe, with evidence for persistent legacy effects that impair post‐drought recovery (Rukh et al. 2023). Drought affects key ecophysiological processes—including carbon assimilation and allocation, water transport, and nutrient dynamics—thereby influencing both tree growth and survival (Gessler et al. 2026). Understanding how trees respond to drought, and which environmental factors control these responses, is therefore critical for predicting forest dynamics under climate change.

Among European tree species, European beech ( *Fagus sylvatica* L.) plays a central ecological and economic role and dominates large areas of temperate forests in Central Europe (Leuschner and Ellenberg 2017). However, increasing drought frequency has raised concerns about its long‐term performance. Severe droughts can cause substantial growth reductions and increased mortality, particularly near the dry margins of the species' distribution (Schuldt et al. 2020; Martinez del Castillo et al. 2022; Klesse et al. 2024). Recent studies identify long‐term climatic water balance as a major driver of beech growth and drought sensitivity, while also showing that responses vary across environmental gradients and are modified by local site conditions such as soil properties, nutrient availability, and stand structure (Grote et al. 2016; Leuschner et al. 2023; Weigel et al. 2023; Gessler et al. 2026).

In addition to environmental drivers, species‐specific traits strongly influence drought responses. Tree species differ in their water‐use strategies, ranging from conservative stomatal regulation to more risk‐prone strategies that maintain gas exchange under declining water availability (McDowell et al. 2008; Klein et al. 2014; Gessler et al. 2026). These are associated with hydraulic traits, carbon allocation, and wood anatomical characteristics (Choat et al. 2012). Diffuse‐porous species such as *Fagus sylvatica* are typically considered to regulate water loss conservatively, whereas ring‐porous species such as oaks (*Quercus* spp.) may maintain higher stomatal conductance but are more vulnerable to hydraulic failure due to their large earlywood vessels (Cochard et al. 1992; Bréda et al. 2006). Similar variation in drought‐response strategies has also been reported among conifers (Bigler et al. 2006; Lévesque et al. 2013; Rigling et al. 2013). Consequently, co‐occurring tree species often exhibit contrasting drought responses (Vanhellemont et al. 2019).

Tree growth responses to drought are commonly described using the concepts of resistance and resilience, which quantify the magnitude of growth reduction during drought and the capacity for post‐drought recovery (Lloret et al. 2011). In addition to growth, drought stress can be recorded in physiological signals preserved in tree rings. Stable carbon isotopes (δ^13^C) provide insights into stomatal conductance and intrinsic water‐use efficiency, reflecting the balance between carbon assimilation and stomatal regulation during photosynthesis (Hubick and Farquhar 1989; McCarroll and Loader 2004). Increased δ^13^C values are typically associated with reduced stomatal conductance under water limitation and have been widely used to infer physiological drought responses (Gessler et al. 2014). Elemental concentrations in tree rings offer complementary information on nutrient transport and stress‐related processes. Calcium (Ca) is particularly informative because its uptake and transport are closely coupled to plant water fluxes: it is transported predominantly in the xylem via the transpiration stream and becomes largely immobile once incorporated into xylem tissue. Consequently, Ca accumulation reflects the combined effects of transpiration‐driven transport processes and plant water use (White and Broadley 2003; Lautner and Fromm 2010; Yin et al. 2022), and has been applied as a dendrochemical indicator of drought responses (Ortega Rodriguez et al. 2023; Papú et al. 2024). However, because Ca concentrations are also influenced by soil Ca availability (White and Broadley 2003) and show pronounced within‐ring variation (Silkin and Ekimova 2012; Gavrikov et al. 2024), the interpretation of tree‐ring Ca as a proxy for drought stress requires caution. Instead, tree‐ring Ca should be interpreted alongside growth and stable isotope indicators to obtain a more comprehensive understanding of physiological drought responses.

Despite increasing research on drought impacts in beech forests, several key uncertainties remain. Although large‐scale studies have demonstrated pronounced spatial variation in drought responses of *Fagus sylvatica* across Europe (Martinez del Castillo et al. 2022; Klesse et al. 2024), knowledge about how climatic, soil, and nutritional factors jointly shape drought responses within regional forest landscapes remains scarce. Moreover, although physiological indicators such as δ^13^C are widely used, their relationship to growth responses across environmental gradients remains poorly resolved. Many studies focus primarily on climatic drivers, whereas soil properties, nutrient availability, and stand characteristics are rarely considered jointly, despite their potential to modulate water availability, nutrition, and carbon allocation under drought (Bréda et al. 2006; Grote et al. 2016; Gessler et al. 2017). In addition, comparative analyses of multiple co‐occurring tree species within the same environmental context remain limited, even though species composition and neighborhood interactions can strongly influence drought responses (Metz et al. 2016).

In this study, we investigated drought resistance and resilience of *Fagus sylvatica* and admixed tree species across 62 forest stands in the Vienna Woods, Austria. Previous studies from the same forest stands focused on species‐specific growth responses (Winter Artusio et al. 2025) or climatic controls of annual growth (Dolschak et al. 2026) using tree‐ring growth records. Here, we integrated these dendrochronological data with newly generated stable carbon isotope (δ^13^C) and tree‐ring calcium measurements as physiological stress indicators, together with a comprehensive set of site, soil, and stand variables to study spatial patterns in drought responses. Specifically, we addressed the following questions: (1) which environmental drivers explain spatial variation in drought‐induced growth responses among *Fagus sylvatica* stands; (2) whether growth responses correspond to physiological stress signals recorded in tree rings; and (3) whether admixed tree species exhibit distinct drought‐response strategies. We hypothesized that drought responses of *Fagus sylvatica* stands are primarily controlled by regional climatic conditions, with secondary modification by site and soil properties; that physiological indicators (δ^13^C and Ca) reflect drought stress but are not necessarily proportional to growth reductions; and that admixed species exhibit contrasting drought‐response strategies due to differences in hydraulic and physiological traits.

## 2 Materials and Methods

### 2.1 Study Area

**FIGURE 1:**
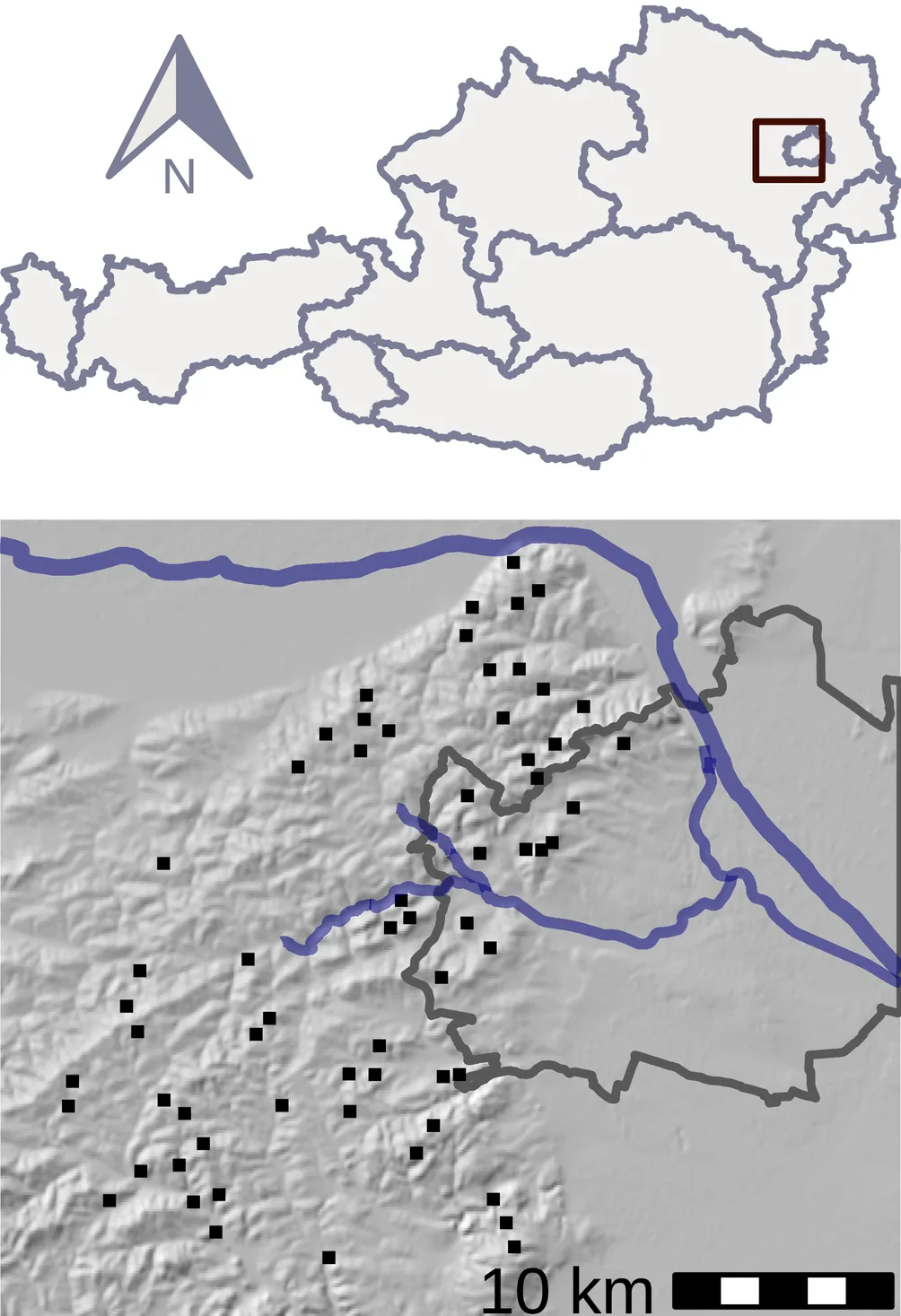
Study area in the Vienna Woods (Wienerwald), Austria and locations of the 62 sampled forest stands (black squares). The gray line indicates the boundary of the city of Vienna. Adapted from Dolschak et al. (2026).

The study was conducted in the Vienna Woods (Wienerwald), a forested landscape adjacent to the city of Vienna in eastern Austria (Figure 1) and part of the UNESCO Biosphere Reserve “Wienerwald.” The region is predominantly underlain by Flysch bedrock, consisting mainly of Tertiary and Mesozoic sandstones and clay‐rich marls. In the southern part, smaller areas are underlain by calcareous substrates derived from the Northern Calcareous Alps (e.g., limestone and dolomite).

The dominant soil types are Stagnosols and Cambisols, with Mull as the prevailing humus form (IUSS Working Group WRB 2022). Elevation ranges from approximately 180 to > 800 m a.s.l., with mean annual precipitation of 600–900 mm and mean annual temperatures of 8°C–9°C.

*Fagus sylvatica* is the dominant tree species throughout the Vienna Woods. In total, 62 beech‐dominated mature forest stands were selected, with an average stand age of 152 years. Several stands contained one or more admixed species, including *Quercus petraea* (*N* = 27), *Quercus cerris* (*N* = 12), *Larix decidua* (*N* = 12), *Pinus nigra* (*N* = 6), *Fraxinus excelsior* (*N* = 4), *Picea abies* (*N* = 4), and *Abies alba* (*N* = 3). Further details are provided in Winter Artusio et al. (2025).

### 2.2 Tree Ring Chronologies and Growth Response to Drought

The 62 forest stands were sampled in autumn 2022. At each site, seven dominant or subdominant healthy trees per species were randomly selected, and two increment cores per tree were extracted at breast height. Trees were distributed across an area of approximately 0.5 ha to ensure comparable site and soil conditions within each stand. Cores were prepared by trimming the transverse surface with a sharp knife and scanned at 1200 dpi using a calibrated Epson Expression 10000XL scanner. Ring widths were measured using WinDendro 2022 (Regent Instruments Inc., Quebec, Canada).

Cross‐dating was performed across all cores per species and across sites, and accuracy was verified using TSAP‐Win (Rinn 2003). Measurement quality was assessed with COFECHA (Holmes 1983). Cores that could not be reliably cross‐dated (e.g., due to rot, breakage, or reaction wood) were excluded. When present, pith offsets were used to estimate tree age. Further methodological details are provided in Winter Artusio et al. (2025). Chronology statistics (Expressed Population Signal, EPS; mean inter‐series correlation, Rbar; and Gleichläufigkeit, GLK) were calculated using the *dplR* package in R (Bunn 2008) and are provided in the Supporting Information (Table S1).

### 2.3 Identification of Drought Events and Growth Metrics

Drought events were identified using stand‐level hydrological modeling for the period 1980–2022. Individual years were evaluated using multiple indicators of drought stress, including transpiration deficit, the number of days with a transpiration index ≤ 0.2 and ≤ 0.3 (indicating strongly reduced transpiration), soil moisture, total transpiration, and stand water balance (see Winter Artusio et al. (2025) and Dolschak et al. (2026)). A 75th percentile threshold was applied to each indicator for annual values and shorter periods within the growing season to identify years with exceptionally severe drought conditions. The selected drought years were further validated by comparing modeled soil moisture during drought years with pre‐ and post‐drought years, wet years, and all remaining years (Winter Artusio et al. 2025). Based on these criteria, four drought years (1983, 2000, 2003, and 2015) were selected for further analyses. To minimize overlapping legacy effects, selected drought years were separated by at least two non‐drought years.

For each tree, ring widths from the 2 years preceding the drought (pre‐drought), the drought year, and the two subsequent years (post‐drought) were used to quantify drought responses. Resistance was defined as the ratio of ring width during drought to mean pre‐drought growth, and resilience as the ratio of post‐drought to pre‐drought growth (Lloret et al. 2011). The recovery metric proposed by Lloret et al. (2011), which quantifies post‐drought growth relative to growth during the drought year, was calculated for completeness and is provided in the Supporting Information (Table S2). However, because the objectives and hypotheses of this study focused specifically on environmental controls of drought resistance and resilience, recovery was not included in the main statistical analyses.

### 2.4 Dendrochemical Analyses

Tree rings corresponding to pre‐drought, drought, and post‐drought periods of each selected drought event were excised under a microscope using cross‐dated reference series. For each stand, species, and sampling period, ring material was pooled and transferred into microtubes to obtain representative stand‐level samples, consistent with the spatial resolution of the environmental variables analyzed (see below).

Aliquots of these samples were digested using HNO_3_ and H_2_O_2_ in a microwave system (Mars 6, CEM, USA). Element concentrations were determined by ICP‐OES (Optima 8300, Perkin Elmer, USA). For the present study, only Ca concentrations were analyzed.

Stable carbon isotope ratios (δ^13^C) were determined for a subset of samples covering two drought periods (1983 and 2015, including pre‐ and post‐drought phases). These two drought events were selected because they represent the earliest and latest severe droughts within the study period, thereby maximizing the temporal separation between events while limiting analytical costs. Subsamples were finely ground, and 1–2 mg of material was weighed into tin capsules and analyzed using an elemental analyzer coupled to an isotope ratio mass spectrometer (EA‐Isolink coupled via ConFlo IV to a Delta V Advantage IRMS; Thermo Scientific). δ^13^C values are expressed in ‰ relative to the Vienna Pee Dee Belemnite (VPDB) standard and were calculated using conventional delta notation. Measurements were calibrated and normalized using international and in‐house reference materials with known δ^13^C values. Analytical precision was within ±0.1‰. Cellulose extraction was not performed because the objective was to compare differences in δ^13^C among pre‐drought, drought, and post‐drought periods rather than analyzing timeseries of absolute δ^13^C values. Although cellulose is commonly preferred for tree‐ring isotope studies, previous studies have demonstrated that whole‐wood δ^13^C provides comparable information for many applications (Borella et al. 1998; Loader et al. 2003; Badea et al. 2021; Helle et al. 2022). Bulk tree‐ring δ^13^C values were therefore used to analyze indicators of physiological responses to drought.

### 2.5 Environmental Variables

Soil sampling was conducted in late summer 2022. At each stand, seven soil cores (5 cm diameter) were taken from the 0–5 cm mineral soil after removal of the organic layer. Deeper samples were collected using a soil auger (2 cm diameter), with four replicates per depth (5–10, 10–20, 20–30, 30–40, and 40–50 cm) pooled for analysis. Two additional large cores (7 cm diameter, 50 cm length) were collected to determine fine soil mass and rock content. Soil sampling covered a representative area of approximately 0.1 ha within the center of each stand. In the laboratory, soils were sieved (< 2 mm). Total nitrogen (N) and sulfur (S) were measured using a LECO SC 444 analyzer (LECO Corporation, MI, USA). Exchangeable Ca, magnesium (Mg), potassium (K), sodium (Na), iron (Fe), and manganese (Mn) were extracted with 1 M ammonium acetate (pH 7; ÖNORM L1086) and analyzed by ICP‐OES (Optima 3000 XL, Perkin Elmer, USA). Soil texture was determined using wet sieving and particle‐size analysis (Sedigraph III, Micromeritics, GA, USA), complemented by manual assessment (ÖNORM L1050). Nutrient stocks (0–50 cm) were calculated from concentrations and fine soil mass. Soil water‐holding capacity (mm m^−2^) was estimated following Gadermaier et al. (2024).

Beech foliage was collected in late summer 2022 from the upper crown of three trees per stand and pooled. Total S was measured using a LECO SC832 analyzer, and total N using a LECO FP928 analyzer (LECO Corporation, MI, USA). Total P, Ca, Mg, and K were determined after HNO_3_/H_2_O_2_ microwave digestion (Mars 6, CEM, USA) by ICP‐OES (Optima 8300, Perkin Elmer, USA).

Stand structure was quantified using angle‐count sampling (Avery and Burkhart 2015) to determine tree density and diameter at breast height. Aboveground woody biomass was estimated using allometric functions (Forrester et al. 2017), and biomass increment was reconstructed from tree‐ring chronologies. Stand vitality was assessed based on crown dieback and classified into three categories: vital, minor stress, and major stress. Stand exposition and slope were measured using a compass and inclinometer, and solar exposure (southness) was derived from these parameters. Elevation was obtained from a digital terrain model.

Meteorological data were derived from the SPARTACUS gridded dataset (Hiebl and Frei 2016) provided by GeoSphere Austria. Each stand was assigned to its corresponding 1 × 1 km grid cell. The dataset provides daily temperature and precipitation records from 1961 to the present.

Further details on sampling and analyses are provided in Berger et al. (2016) and Mayer et al. (2025).

### 2.6 Statistical Analysis

Drought resistance and resilience were calculated following Lloret et al. (2011), as the ratio of drought growth or post‐drought growth to pre‐drought growth (see also above). Changes in δ^13^C and Ca concentrations were expressed as absolute differences between pre‐drought, drought, and post‐drought phases.

**FIGURE 2:**
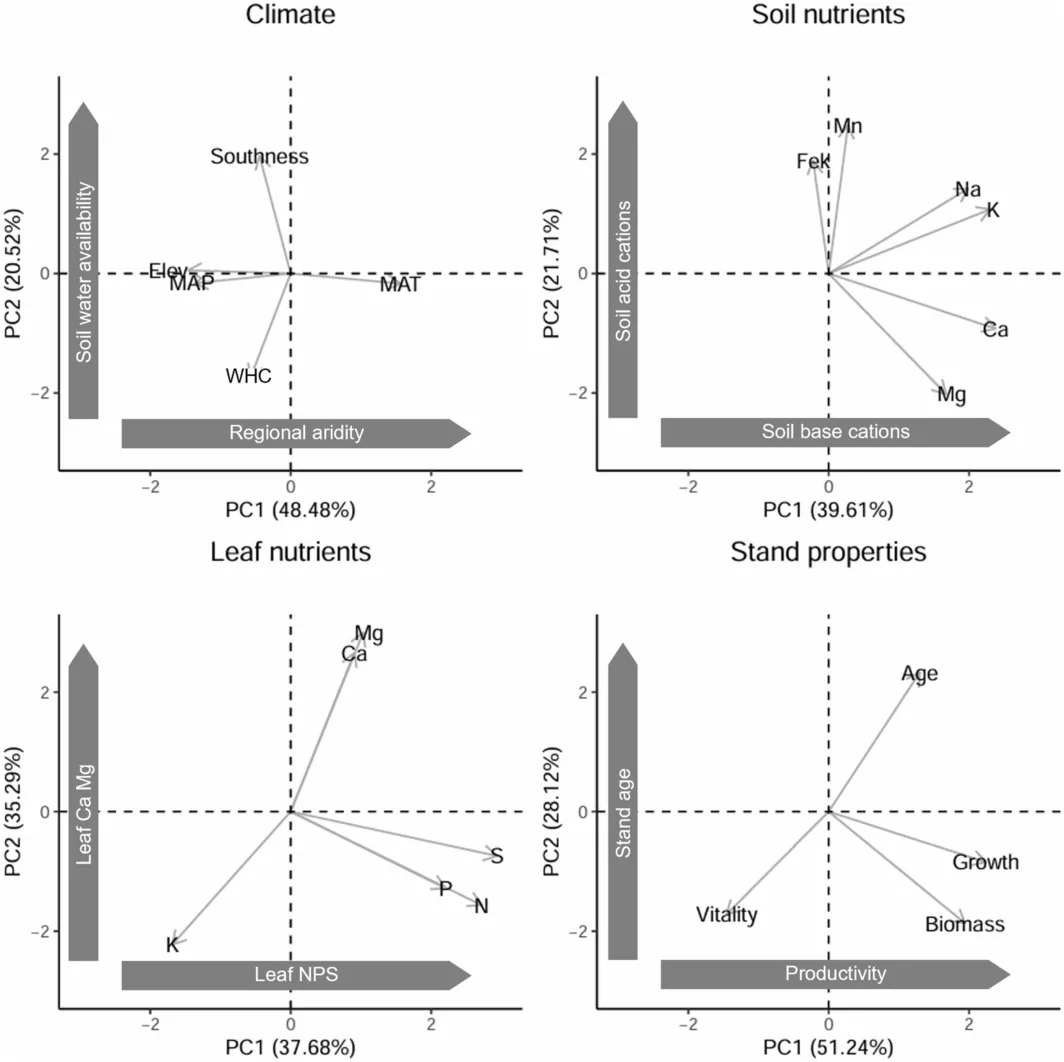
Principal component analyses (PCA) of climate variables, soil chemical properties, leaf nutrient concentrations, and stand properties across 62 European beech ( *Fagus sylvatica* ) stands in the Vienna Woods, Austria. The first and second axes of each PCA were used as composite environmental variables in subsequent analyses. Climate variables—Elev, elevation (m a.s.l.); MAP, mean annual precipitation (mm); MAT, mean annual temperature (°C); WHC, soil water‐holding capacity (mm m^−2^). Soil cations—stocks of Fe (iron), Mn (manganese), Na (sodium), K (potassium), Ca (calcium), and Mg (magnesium) (g m^−2^). Leaf nutrients—concentrations of P (phosphorus), S (sulfur), N (nitrogen), K, Ca, and Mg (mg g^−1^). Stand properties—Age, stand age (years); Vitality, crown vitality index (unitless); Biomass, aboveground biomass (tC ha^−1^); Growth, aboveground biomass increment (tC ha^−1^ year^−1^).

To reduce multicollinearity among explanatory variables and to identify broad environmental gradients rather than individual predictor variables, principal component (PC) analyses were performed separately for climate variables, soil nutrients, leaf nutrients, and stand properties (Figure 2). The first two principal components of each PC analysis were used as composite environmental variables in subsequent analyses. For climate, PC1 represented a regional aridity gradient, describing a transition from cool, moist, high‐elevation sites to warm, dry, low‐elevation sites, whereas PC2 primarily reflected a soil water availability gradient associated with soil water‐holding capacity and solar exposure (southness). For soil nutrients, PC1 represented a soil base cation gradient (Ca, Mg, K, and Na), whereas PC2 represented a soil acid cation gradient (Fe and Mn). For leaf nutrients, PC1 represented a gradient of foliar N, P, and S concentrations, whereas PC2 represented a gradient of foliar Ca and Mg concentrations, with K loading in the opposite direction. For stand properties, PC1 represented a stand productivity gradient (growth and biomass), whereas PC2 primarily represented a stand age gradient (stand age and vitality). Pearson correlation analyses were performed between PC scores and the original measured site, soil, foliar, and stand variables to facilitate the interpretation of the environmental gradients.

Prior to statistical analysis, dendrochronological and dendrochemical variables were averaged at the stand and species level for each drought event because the environmental variables (e.g., soil properties, foliar nutrient concentrations, stand characteristics) were available only at the stand level. Consequently, all analyses were conducted at this spatial resolution to ensure consistency between response and explanatory variables. The objective of this study was to identify spatial variation in drought responses across environmental gradients rather than differences among individual drought events. Therefore, stand‐level drought responses were subsequently averaged across the four selected drought events to emphasize consistent spatial patterns over event‐specific variability. As a sensitivity analysis, linear mixed‐effects models were used to test whether the relationships between drought responses and environmental gradients differed among drought events. We compared models including environmental variables and drought event as additive fixed effects and stand as a random effect with models additionally including environmental variable × drought event interactions. The additive models consistently provided a better fit, as indicated by lower AIC values (Table S3), suggesting that relationships between environmental gradients and drought responses were broadly consistent across drought events and supporting the use of pooled stand‐level responses. Temporal variability in drought responses has been addressed in previous studies (Winter Artusio et al. 2025; Dolschak et al. 2026).

Linear regression models were used to identify environmental variables significantly related to drought responses. These variables were subsequently incorporated into a structural equation model to evaluate direct and indirect relationships while accounting for correlations among predictor variables (Beaujean 2014). The initial structural equation model included all variables identified as significant in regression analyses. Model simplification was performed by stepwise removal of nonsignificant paths, retaining only relationships with *p* < 0.05.

Linear regression models were used to assess relationships between changes in tree‐ring growth, δ^13^C, and Ca during drought and post‐drought periods. One‐sample Student's *t*‐tests were used to test whether response variables differed from 0.

Differences in drought responses between *Fagus sylvatica* and admixed tree species were assessed using paired comparisons at the site level, restricting analyses to sites where both species co‐occurred. One‐sample Student's *t*‐tests were used to test whether response variables differed from reference values (1 for tree‐ring width and 0 for changes in δ^13^C and Ca concentrations). In addition, mean response, unstandardized effect size (mean deviation from the reference value), and its 95% confidence interval were calculated.

All analyses were conducted in R (R Core Team 2024), using the lavaan package for SEM (Rosseel 2012), nlme for linear mixed‐effects models (Pinheiro et al. 2014), and ggplot2 for visualization (Wickham 2016). The significance level was set at *p* < 0.05.

## 3 Results

### 3.1 Environmental Controls on Drought Resistance and Resilience

**FIGURE 3:**
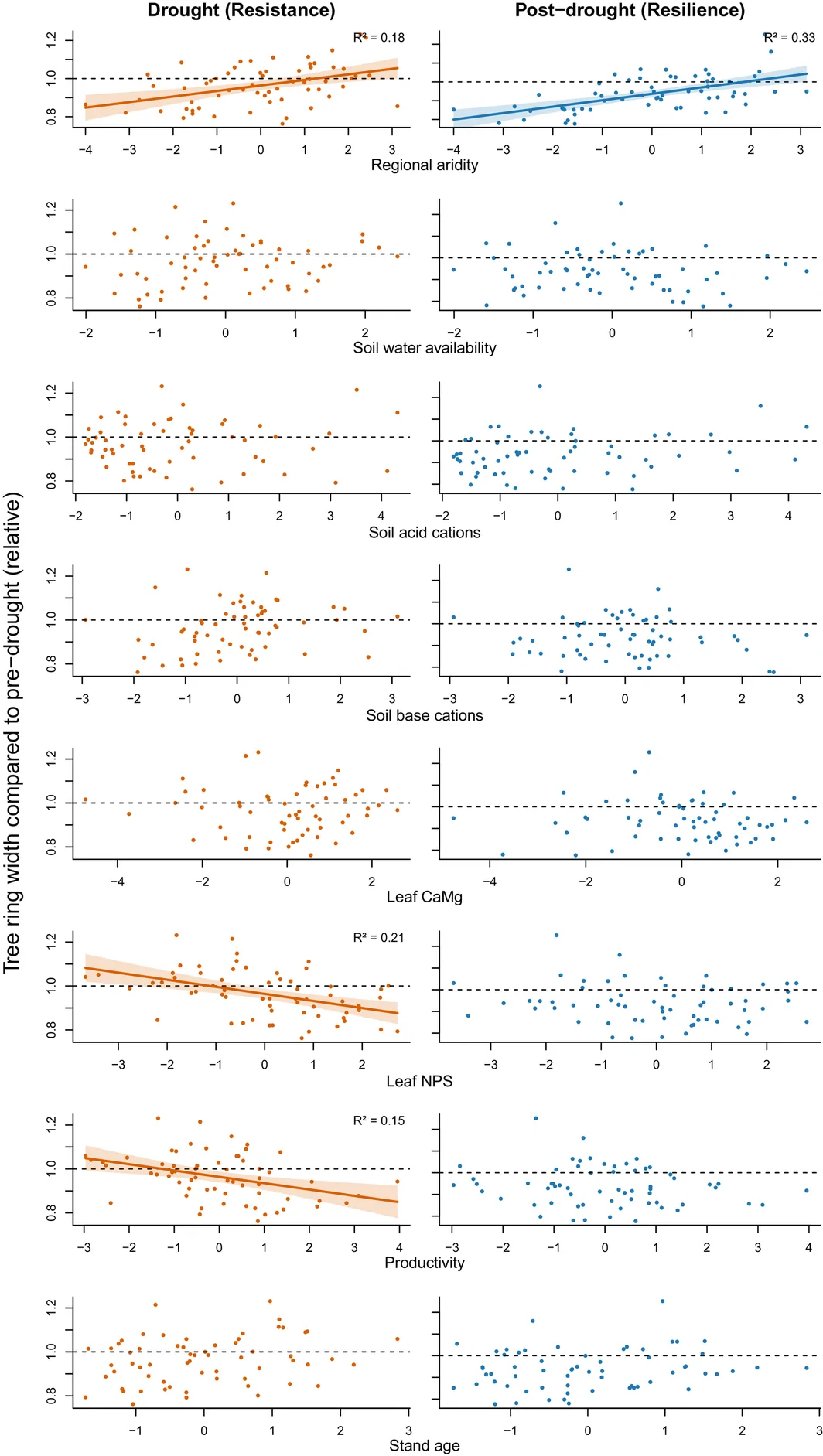
Relationships between relative tree‐ring width during drought (resistance) and after drought (resilience) and composite environmental variables derived from the first and second axes of principal component analyses (see Figure 2). Points represent site‐level mean values from 62 European beech ( *Fagus sylvatica* ) stands in the Vienna Woods, Austria, across four drought events (1983, 2000, 2003, and 2015). Solid lines indicate fitted linear relationships, with shaded areas representing 95% confidence intervals. Tree‐ring width is expressed relative to pre‐drought conditions. Only significant relationships (*p* < 0.05) are shown. The horizontal dashed line indicates no change relative to pre‐drought conditions; values above the line indicate increased growth, whereas values below the line indicate reduced growth.

Tree growth responses of *Fagus sylvatica* to drought were significantly related to environmental gradients derived from scores of separate principal component analyses (Figure 3). Drought resistance increased with climate PC1 scores (regional aridity gradient). Resistance decreased significantly with leaf nutrients PC1 scores (leaf NPS gradient) and stand properties PC1 scores (stand productivity gradient).

Post‐drought resilience was positively related to climate PC1 scores (regional aridity gradient). Many sites exhibited resilience values below pre‐drought levels, indicating incomplete recovery within 2 years after drought. No significant relationships (*p* > 0.05) were observed between resistance or resilience and climate PC2 scores (soil water availability gradient), soil nutrients PC1 scores (soil base cation gradient), soil nutrients PC2 scores (soil acid cation gradient), leaf nutrients PC2 scores (leaf CaMg gradient), or stand properties PC2 scores (stand age gradient).

**FIGURE 4:**
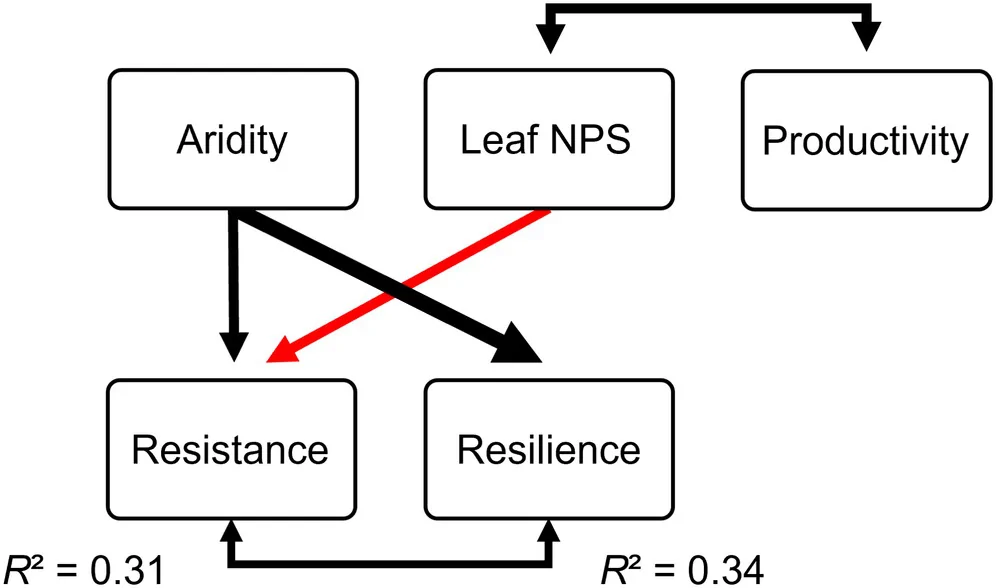
Structural equation model illustrating the effects of composite environmental variables on relative tree‐ring width during drought (resistance) and after drought (resilience) in 62 European beech ( *Fagus sylvatica* ) stands in the Vienna Woods, Austria. Composite variables were derived from the first and second axes of principal component analyses of environmental variables (see Figure 2). Single‐headed arrows indicate significant positive (black) and negative (red) effects, respectively, and double‐headed arrows indicate significant positive correlations among variables. Arrow thickness reflects the strength of standardized path coefficients. A positive effect of aridity on resistance indicates smaller reductions in tree‐ring width during drought at more arid sites.

The structural equation model supported these relationships (Figure 4). Climate PC1 scores (regional aridity gradient) had significant positive effects on both resistance and resilience, whereas leaf nutrients PC1 scores (leaf NPS gradient) had a negative effect on resistance but no direct effect on resilience. Resistance and resilience were positively related. Stand properties PC1 (stand productivity gradient) were positively correlated with leaf nutrients PC1 scores (leaf NPS gradient), but no significant effect on resistance or resilience was found. The model explained 31% of the variance in resistance and 34% of the variance in resilience.

### 3.2 Relationships Between Growth Responses, Isotopic Composition, and Tree‐Ring Calcium

**FIGURE 5:**
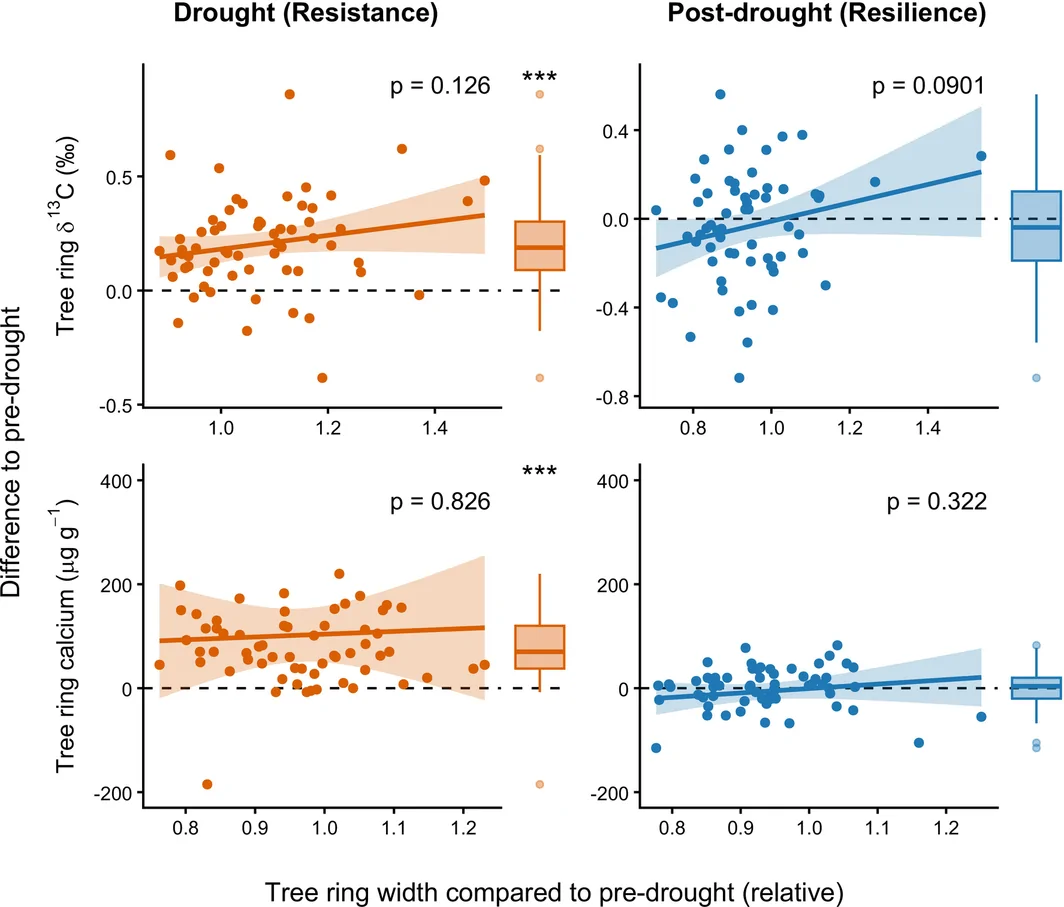
Relationships between relative tree‐ring width during drought (resistance) and after drought (resilience) and absolute changes in tree‐ring δ^13^C (‰) and calcium (Ca) concentrations (μg g^−1^) relative to pre‐drought conditions. Points represent site‐level mean values from 62 European beech ( *Fagus sylvatica* ) stands in the Vienna Woods, Austria. Solid lines indicate fitted linear relationships, with shaded areas representing 95% confidence intervals. The horizontal dashed line indicates no change relative to pre‐drought conditions. Boxplots summarize changes in δ^13^C and Ca during and after drought relative to pre‐drought conditions. Asterisks indicate significant differences from zero (***p < 0.001). Mean δ^13^C values and corresponding growth responses were calculated from the drought years 1983 and 2015, whereas Ca concentrations and associated growth responses were calculated from the drought years 1983, 2000, 2003, and 2015, respectively.

Changes in tree‐ring δ^13^C and Ca concentrations of *Fagus sylvatica* were not significantly related to growth responses (Figure 5). During drought, δ^13^C values increased significantly relative to pre‐drought conditions but were not related to resistance. Similarly, tree‐ring Ca concentrations increased during drought but showed no significant relationship with resistance.

Following drought, δ^13^C changes were not related to resilience, although a weak positive trend was observed (*p* = 0.09). Post‐drought δ^13^C values did not differ significantly from pre‐drought levels. Likewise, tree‐ring Ca concentrations showed no significant relationship with resilience and did not differ significantly from pre‐drought conditions.

### 3.3 Species Differences in Drought Responses

**FIGURE 6:**
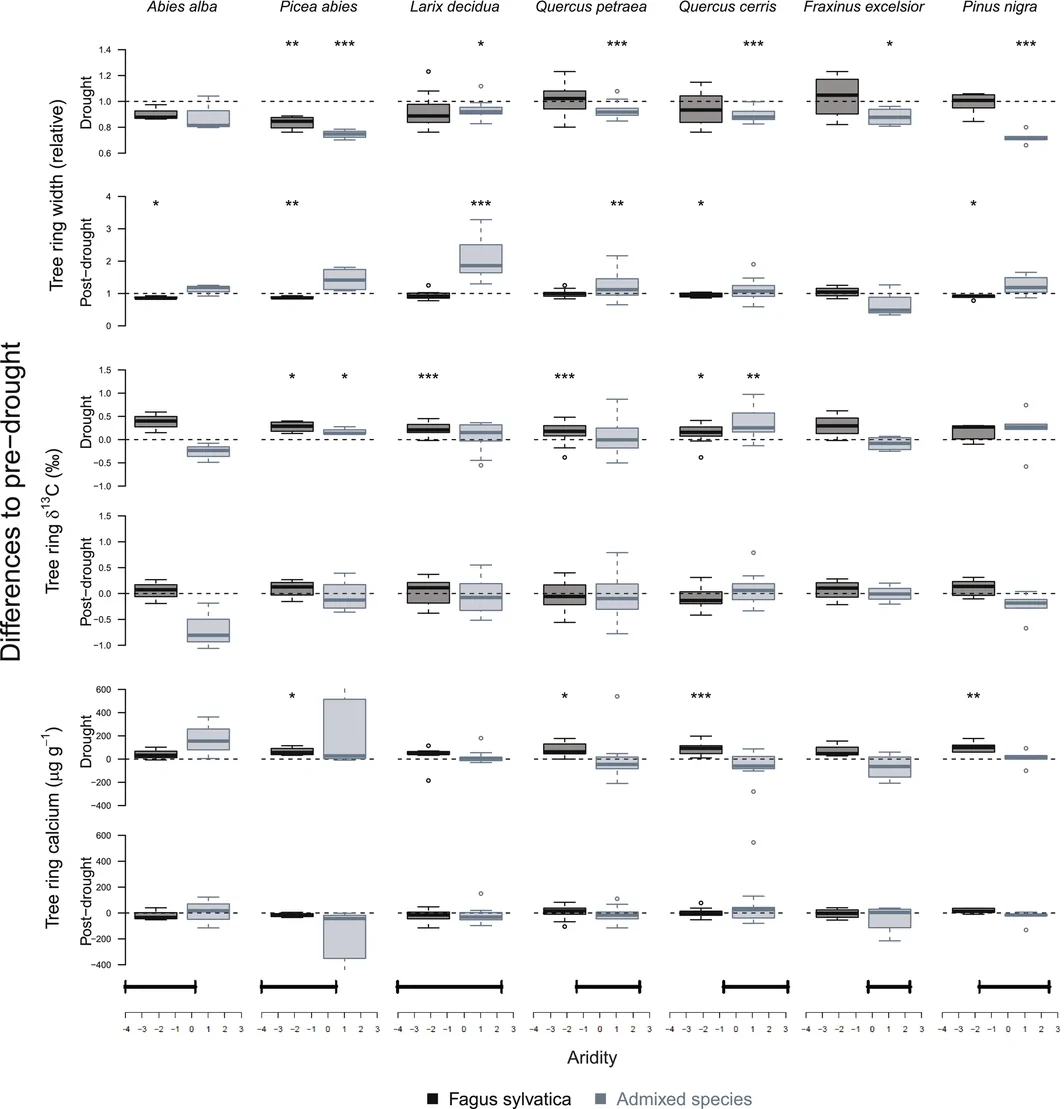
Differences in drought responses between European beech ( *Fagus sylvatica* ) and admixed tree species across study sites. Boxplots show changes in relative tree‐ring width, tree‐ring δ^13^C (‰), and calcium (Ca) concentrations (μg g^−1^) during and after drought relative to pre‐drought conditions. Because admixed species occurred only at subsets of sites, comparisons were restricted to sites where the respective species co‐occurred with *Fagus sylvatica* . For tree‐ring width, the horizontal dashed line indicates no change relative to pre‐drought conditions (value = 1); for δ^13^C and Ca, it indicates no change (value = 0). Tree‐ring width and Ca concentrations were analyzed for four drought events (1983, 2000, 2003, and 2015), whereas δ^13^C was analyzed for two drought events (1983 and 2015). Asterisks indicate significant differences from the respective reference values (* *p* < 0.05, ** *p* < 0.01, *** *p* < 0.001). Horizontal bars at the bottom of the figure indicate the occurrence range of admixed species along the regional aridity gradient.

Comparisons between *Fagus sylvatica* and admixed tree species revealed pronounced interspecific variation in drought responses (Figure 6, Table S4). Because admixed species occurred only at subsets of sites, comparisons were restricted to sites where the respective species co‐occurred with *Fagus sylvatica* .

The distribution of admixed species along the aridity gradient differed among species. *Abies alba* and *Picea abies* occurred mainly at cooler and moister sites, whereas *Quercus cerris* , *Quercus petraea* , *Fraxinus excelsior* , and *Pinus nigra* were more frequent at more arid sites. *Larix decidua* occurred across most of the gradient (Figure 6 bottom).

During drought, several admixed species showed significant reductions in tree‐ring width relative to pre‐drought conditions, including *Picea abies* , *Quercus petraea* , *Quercus cerris* , *Fraxinus excelsior* , and *Pinus nigra* , whereas *Abies alba* did not exhibit a significant growth reduction. Growth reductions in *Fagus sylvatica* were generally moderate compared with those of the admixed species.

Following drought, most admixed species recovered to pre‐drought growth levels or showed overcompensation, with post‐drought growth either not significantly different from 1 or significantly > 1. In contrast, *Fagus sylvatica* showed weaker recovery at some sites, with post‐drought growth remaining close to or slightly below pre‐drought levels.

Isotopic responses differed among species. During drought, significantly higher tree‐ring δ^13^C values were observed for *Picea abies* and *Quercus cerris* . At these sites, *Fagus sylvatica* also showed significantly higher δ^13^C values relative to pre‐drought conditions. In contrast, at sites where *Larix decidua* and *Quercus petraea* occurred, only *Fagus sylvatica* exhibited significantly higher δ^13^C values during drought. Post‐drought δ^13^C values did not differ significantly from pre‐drought conditions for any species.

Tree‐ring Ca responses also differed among species. During drought, significantly higher Ca concentrations were observed only in *Fagus sylvatica* at sites where *Picea abies* , *Quercus petraea* , *Quercus cerris* , and *Pinus nigra* occurred, whereas no significant changes were detected for the admixed species. Post‐drought Ca concentrations did not differ significantly from pre‐drought conditions for any species.

## 4 Discussion

Drought responses of forest stands emerge from the interaction of long‐term environmental conditions, physiological stress responses, and species‐specific strategies. Here, we examined how environmental gradients shape drought resistance and resilience of *Fagus sylvatica* , whether growth responses correspond to physiological stress signals recorded in tree rings, and how admixed tree species differ in their drought‐response strategies. Our results identified regional climate as an important driver of growth responses of *Fagus sylvatica* in the Vienna Woods. Relationships with stand productivity and foliar nutrient status further indicate that factors beyond climate contribute to drought responses. In contrast, physiological signals recorded in tree rings (δ^13^C and Ca) showed clear drought responses but were not related to spatial variation in growth reductions, consistent with a potential decoupling between physiological responses and growth dynamics. In addition, admixed tree species exhibited contrasting drought‐response strategies, highlighting the importance of species‐specific physiological mechanisms.

### 4.1 Environmental Controls on Drought Resistance and Resilience

Drought resistance and resilience of *Fagus sylvatica* stands increased along the regional aridity gradient. This indicates that sites characterized by warmer, drier conditions and lower elevation generally exhibited smaller growth reductions and more rapid post‐drought recovery than cooler, moister sites at higher elevations. Although this pattern may appear counterintuitive, similar responses have been reported across forest ecosystems and are commonly attributed to long‐term acclimation or adaptation to prevailing climatic conditions (Bréda et al. 2006; Choat et al. 2012; Gessler et al. 2026). Trees growing under chronically dry conditions may develop structural and physiological traits that enhance drought tolerance, including deeper rooting systems, modified hydraulic architecture, and more conservative stomatal regulation. In contrast, trees at cooler and moister sites are less frequently exposed to water limitation and may therefore be more vulnerable to extreme drought events. This is consistent with the widespread occurrence of incomplete recovery within 2 years after drought, particularly at sites at higher elevations with a cooler and moister climate. Such patterns point to drought legacy effects, which have been widely documented and are often linked to hydraulic damage, altered carbon allocation, or depletion of carbon reserves (Rukh et al. 2023; Gessler et al. 2026).

In addition to climate, drought resistance was negatively related to both the leaf NPS and stand productivity gradients, indicating larger growth reductions in nutrient‐rich and more productive stands. Gessler et al. (2017) proposed that high nutrient availability may predispose trees to drought damage by promoting greater aboveground biomass, larger crowns, higher shoot‐to‐root ratios, and increased transpirational demand, thereby reducing hydraulic safety margins. Consistent with this framework, Mausolf et al. (2018) reported a trade‐off between growth and drought tolerance in European beech, showing that trees with larger crowns and higher radial growth experienced stronger drought‐induced growth reductions, which they attributed to increased canopy water demand. Likewise, Xu et al. (2020) identified ecosystem productivity as a major predictor of drought sensitivity across North American forests. The positive correlation between leaf NPS and stand productivity gradients in the structural equation model supports this interpretation, suggesting that foliar nutrient concentrations primarily reflect a productive, resource‐acquisitive stand strategy rather than a direct physiological effect of nutrients on drought resistance. In this context, higher nutrient availability may facilitate rapid growth under favorable conditions while simultaneously increasing vulnerability to water limitation during severe drought, consistent with the conceptual framework proposed by Gessler et al. (2017).

With our principal component approach, we aimed to reduce multicollinearity among environmental variables and to identify broad environmental gradients rather than using individual variables that maximized predictive performance. This approach sacrifices some predictive power in favor of a more generalized representation of the environmental conditions across the study area. Accordingly, the structural equation model explained about one third of the observed variation, with the regional climate gradient emerging as the strongest predictor of both drought resistance and resilience.

Part of the remaining variability likely reflects environmental and biological factors that were not explicitly captured in the present study. Although soil properties were assessed to a depth of 50 cm, deeper soil layers may also contribute to tree water uptake at some sites. However, recent evidence suggests that European beech does not substantially compensate for declining topsoil water availability by increasing water uptake from deeper soil layers during drought (Gessler et al. 2022). Nevertheless, site‐ and species‐specific differences in rooting architecture and root distribution could still influence access to available soil water and thereby contribute to drought responses (Braun et al. 2005; Meier et al. 2018). Genetic variation among individuals and populations, as well as differences in stand history and legacy effects, may also contribute to variation in drought tolerance and resilience (Depardieu et al. 2020; Pretzsch 2022; Gazol et al. 2023). In addition, soil conditions and nutrient availability have changed across the study area since the 1980s (Mayer et al. 2025). Consequently, the environmental variables used in this study represent present‐day conditions and may not fully reflect the site conditions under which the historical drought responses developed. Likewise, the absence of significant relationships with the soil variables should not be interpreted as evidence that belowground processes are unimportant. Rather, soil‐related effects may have been partly masked by covariance with broader environmental gradients (Figure S1). Nevertheless, the significant relationships between stand productivity, foliar nutrient status, and drought resistance indicate that resource availability remains important determinants of drought responses, even though their individual effects could not be disentangled in the present study. Consequently, the relative importance of climatic and edaphic controls may differ in landscapes where climate and soil properties are less tightly coupled.

### 4.2 Physiological Signals of Drought Stress

We expected drought‐induced growth reductions to be associated with physiological stress signals recorded in tree‐ring δ^13^C and Ca concentrations. Although both parameters responded strongly to drought, neither explained spatial variation in growth responses. During drought, δ^13^C values increased significantly, indicating reduced stomatal conductance and increased intrinsic water‐use efficiency (Hubick and Farquhar 1989; McCarroll and Loader 2004). However, this response occurred consistently across sites, regardless of the magnitude of growth reductions, suggesting that drought triggered a widespread physiological adjustment in stomatal regulation that was not directly proportional to growth decline.

Tree‐ring Ca concentrations also increased during drought but showed no relationship with growth responses. Because Ca transport is largely driven by the transpiration stream, reduced water flux would be expected to limit Ca supply to developing tissues. The observed increase therefore indicates that additional processes offset or overcompensate this limitation. One plausible explanation is that drought‐induced soil water depletion increases solute concentrations in the soil solution, including Ca, thereby enhancing concentration gradients between soil and plant and partially compensating for reduced mass flow. In addition, reduced radial growth during drought may concentrate Ca within narrower tree rings, further amplifying apparent increases in Ca concentrations. Together, these findings indicate that tree‐ring Ca integrates multiple processes—including transpiration‐driven transport, soil solution chemistry, and growth dynamics—and therefore does not provide a simple proxy for plant water use or growth responses.

The interpretation of the δ^13^C and Ca results should also be considered in light of several methodological limitations. First, dendrochemical analyses were based on pooled ring material from all sampled trees within each stand and sampling period to obtain stand‐level estimates, reducing biological variability among individual trees. Second, isotopic measurements were available only for two drought events (1983 and 2015), selected because they represent the earliest and latest severe droughts within the study period and thus maximize temporal separation while limiting analytical effort. Consequently, the results should not be extrapolated to all drought events investigated in this study. Nevertheless, drought resulted in pronounced increases in δ^13^C despite comparatively moderate growth reductions, indicating that physiological stress signals can occur even when growth reductions are relatively small. This likely reflects differences in temporal integration: δ^13^C captures short‐term physiological responses during periods of carbon assimilation, whereas radial growth integrates environmental conditions over the entire growing season (McCarroll and Loader 2004). In addition, radial growth reflects whole‐tree carbon balance, including photosynthesis, respiration, carbon allocation, and the mobilization of stored reserves, such that changes in stomatal conductance inferred from δ^13^C do not necessarily translate directly into growth responses. Third, δ^13^C was determined from whole wood rather than extracted cellulose, which is considered to provide a more robust indicator of physiological stress (Helle et al. 2022). However, previous studies have shown that whole‐wood δ^13^C provides comparable information for many applications (Borella et al. 1998; Loader et al. 2003). For Ca, interpretation is further complicated by pronounced within‐ring variation, with concentrations typically decreasing from earlywood toward latewood (Silkin and Ekimova 2012; Gavrikov et al. 2024), such that bulk tree‐ring measurements integrate seasonal variation in Ca incorporation.

Both δ^13^C and Ca consistently returned to pre‐drought levels, whereas radial growth incompletely recovered at many sites. This pattern is consistent with a potential decoupling between short‐term physiological adjustments and longer‐term growth dynamics. However, given the methodological limitations of the physiological datasets, this interpretation should be treated with caution.

### 4.3 Species‐Specific Drought‐Response Strategies

Comparisons between *Fagus sylvatica* and admixed tree species revealed pronounced interspecific differences in drought responses. Several admixed species showed stronger growth reductions during drought but generally recovered more rapidly afterwards. This emphasizes that drought tolerance depends on the balance between growth reduction and post‐drought recovery.

The combined patterns of growth, δ^13^C, and Ca responses indicate distinct drought‐response strategies among species. *Fagus sylvatica* showed moderate growth reductions accompanied by pronounced increases in δ^13^C and Ca, consistent with strong stomatal regulation and reduced transpiration. This conservative strategy likely protects the hydraulic system during drought but limits carbon assimilation and contributes to relatively weak post‐drought recovery (Leuschner 2020; Pretzsch et al. 2020; Leuschner et al. 2023).

In contrast, *Quercus* species exhibited stronger growth reductions but weaker isotopic and Ca responses, suggesting less pronounced stomatal limitation and continued gas exchange under drought conditions. This pattern is consistent with ring‐porous species relying on stored carbohydrates and earlywood formation, which can sustain growth but increase vulnerability to hydraulic dysfunction (McDowell et al. 2008; Choat et al. 2012; Michelot et al. 2012; Klein et al. 2014). Their comparatively strong recovery indicates a capacity to compensate for drought‐induced growth reductions when favorable conditions return.

*Fraxinus excelsior* showed comparatively weak recovery despite being considered drought‐tolerant, possibly reflecting additional stress factors such as ash dieback caused by *Hymenoscyphus fraxineus* (McKinney et al. 2014).

Among conifers, *Pinus nigra* and *Larix decidua* showed strong growth reductions during drought but rapid recovery afterwards, with *Larix decidua* even exhibiting overcompensation. This suggests a strategy of temporary growth suppression followed by compensatory growth, potentially linked to flexible carbon allocation and access to deeper soil water (Rossi et al. 2009; Sánchez‐Salguero et al. 2013; Vennetier et al. 2015). In contrast, *Picea abies* showed strong growth reductions accompanied by increased δ^13^C, indicating pronounced stomatal closure and reduced carbon assimilation. As a shallow‐rooted, isohydric species, it rapidly limits water loss under drought, which protects hydraulic integrity but constrains growth (Zang et al. 2014; Brinkmann et al. 2016). *Abies alba* showed little or no growth reduction, indicating relatively high drought tolerance. This is consistent with its deep rooting system and early stomatal regulation, which enable effective drought avoidance as long as deep soil water is accessible (Vitasse et al. 2019; Walder et al. 2021). However, particularly *Picea abies* and *Abies alba* were represented by only a few stands. Consequently, species‐specific comparisons for these taxa should be interpreted with caution, and future studies including larger sample sizes are needed to confirm these patterns.

Our findings demonstrate that co‐occurring species differ not only in the magnitude of growth responses but also in the physiological pathways through which drought responses are expressed. The coexistence of species with contrasting strategies—from conservative regulation in *Fagus sylvatica* to compensatory growth or avoidance in other species—may enhance the stability of mixed forests under increasing drought frequency through complementary responses to water limitation (Metz et al. 2016; Gessler et al. 2026).

### 4.4 Synthesis

Taken together, our results demonstrate that drought responses of *Fagus sylvatica* are driven by environmental gradients, with the regional climate gradient emerging as an important predictor of both drought resistance and resilience. At the same time, the relationships between foliar nutrient status, stand productivity, and drought resistance indicate that resource availability also contributes to drought responses, although their individual effects could not be fully disentangled from the broader environmental gradients in the Vienna Woods. Physiological indicators revealed widespread drought‐induced stress responses that were not reflected in radial growth, a pattern consistent with a potential decoupling between short‐term physiological adjustments and longer‐term growth dynamics. Pronounced differences among co‐occurring species further emphasize that drought responses are shaped by contrasting resource‐use strategies. Together, these findings demonstrate that forest responses to drought emerge from the interaction of climate, site conditions, physiology, and species composition, and suggest that mixed forests comprising species with contrasting drought‐response strategies may enhance ecosystem resilience under future drought regimes.

## Author Contributions

**Mathias Mayer:** conceptualization, methodology, data curation, formal analysis, validation, visualization, writing – original draft, writing – review and editing, investigation. **Klaus Dolschak:** methodology, validation, formal analysis, data curation, visualization, writing – review and editing, writing – original draft. **Emilia Winter Artusio:** methodology, data curation, investigation, validation, formal analysis, writing – review and editing, writing – original draft. **Michael Grabner:** writing – review and editing, conceptualization, methodology, data curation, investigation, validation, formal analysis, supervision, resources, writing – original draft. **Michael Tatzber:** methodology, investigation, validation, data curation, formal analysis, writing – review and editing, resources, writing – original draft. **Iftekhar U. Ahmed:** methodology, data curation, investigation, validation, formal analysis, writing – review and editing, writing – original draft. **Elisabeth Wächter:** methodology, validation, writing – review and editing, investigation, formal analysis, data curation, writing – original draft. **Isolde K. Berger:** methodology, formal analysis, data curation, writing – review and editing, investigation, writing – original draft. **Pétra Berger:** methodology, writing – review and editing, formal analysis, data curation, investigation, writing – original draft. **Wolfgang Wanek:** conceptualization, methodology, data curation, investigation, validation, formal analysis, supervision, project administration, resources, writing – review and editing, writing – original draft. **Torsten W. Berger:** conceptualization, methodology, data curation, investigation, validation, formal analysis, supervision, resources, project administration, funding acquisition, writing – original draft, writing – review and editing.

## Funding

This work was supported by the Federal Ministry of Agriculture, Forestry, Regions and Water Management, (WF‐Research project, number 101725, “WiwaKonKlim”), and the Austrian Science Fund (FWF, Grant‐DOI: 10.55776/P23861).

## Conflicts of Interest

The authors declare no conflicts of interest.

## Supporting information


**Table S1:** Summary statistics of dendrochronologies of tree‐ring width for each tree species. Reported are the minimum values, mean, and standard deviation (SD) of the mean inter‐series correlation (Rbar), Expressed Population Signal (EPS), and Gleichläufigkeit (GLK), calculated separately for each study site.
**Table S2:** Mean drought resistance, resilience, and recovery (Lloret et al. 2011) for stand‐level tree ring width *Fagus sylvatica* and admixed tree species across all study sites. Recovery is provided for completeness and to facilitate comparison with previous studies but was not included in the main analyses.
**Table S3:** Comparison of additive and interaction linear mixed‐effects models testing whether relationships between environmental gradients and drought responses of stand‐level tree ring width differed among the four drought events. Models included drought event and each environmental gradient as additive fixed effects or with an additional environmental gradient × drought event interaction, with stand included as a random effect. Lower AIC values indicate better model fit. For all environmental gradients, additive models were consistently preferred over interaction models, supporting the pooling of drought responses across events.
**Table S4:** Mean drought‐response values for relative tree‐ring width, tree‐ring δ^13^C (‰), and calcium (Ca) concentrations (μg g^−1^), together with unstandardized effect sizes (mean − reference value), their 95% confidence intervals (CI), and one‐sample *t*‐test *p*‐values for *Fagus sylvatica* and each admixed tree species. Statistics were calculated independently for each species using only the sites where the respective admixed species co‐occurred with *Fagus sylvatica* . Reference values were 1 for relative tree‐ring width and 0 for changes in δ^13^C and Ca concentrations. Effect sizes therefore represent deviations from pre‐drought conditions rather than differences between tree species.
**Figure S1:** Pearson correlation coefficients between the principal component (PC) scores representing the environmental gradients used in the analyses and the original measured environmental variables. The correlation matrix illustrates how the composite variables derived from principal component analysis represent the underlying climate, soil, foliar nutrient, and stand variables. Positive correlations are shown in blue and negative correlations in red, with color intensity proportional to the correlation coefficient.

## Data Availability

The data that support the findings of this study are openly available in EDI Data Portal at 10.6073/pasta/cfb899c74237bf059b3e95bb2f20733e.
